# Virtual Clinical and Precision Medicine Tumor Boards—Cloud-Based Platform–Mediated Implementation of Multidisciplinary Reviews Among Oncology Centers in the COVID-19 Era: Protocol for an Observational Study

**DOI:** 10.2196/26220

**Published:** 2021-09-10

**Authors:** Livio Blasi, Roberto Bordonaro, Vincenzo Serretta, Dario Piazza, Alberto Firenze, Vittorio Gebbia

**Affiliations:** 1 Azienda di Rilievo Nazionale ad Alta Specializzazione Civico Palermo Italy; 2 Azienda di Rilievo Nazionale ad Alta Specializzazione Garibaldi Catania Italy; 3 Gruppo Studio Tumori Urologici Foundation for Cancer Research Palermo Italy; 4 University of Palermo Palermo Italy; 5 La Maddalena Cancer Center Palermo Italy

**Keywords:** virtual tumor board, multidisciplinary collaboration, oncology, multidisciplinary communication, health services, multidisciplinary oncology consultations, virtual health, digital health, precision medicine, tumor, cancer, cloud-based, platform, implementation, oncology, COVID-19

## Abstract

**Background:**

Multidisciplinary tumor boards play a pivotal role in the patient-centered clinical management and in the decision-making process to provide best evidence-based, diagnostic, and therapeutic care to patients with cancer. Among the barriers to achieve an efficient multidisciplinary tumor board, lack of time and geographical distance play a major role. Therefore, the elaboration of an efficient virtual multidisciplinary tumor board (VMTB) is a key point to successfully obtain an oncology team and implement a network among health professionals and institutions. This need is stronger than ever during the COVID-19 pandemic.

**Objective:**

This paper presents a research protocol for an observational study focused on exploring the structuring process and the implementation of a multi-institutional VMTB in Sicily, Italy. Other endpoints include analysis of cooperation between participants, adherence to guidelines, patients’ outcomes, and patient satisfaction.

**Methods:**

This protocol encompasses a pragmatic, observational, multicenter, noninterventional, prospective trial. The study’s programmed duration is 5 years, with a half-yearly analysis of the primary and secondary objectives’ measurements. Oncology care health professionals from various oncology subspecialties at oncology departments in multiple hospitals (academic and general hospitals as well as tertiary centers and community hospitals) are involved in a nonhierarchic manner. VMTB employs an innovative, virtual, cloud-based platform to share anonymized medical data that are discussed via a videoconferencing system both satisfying security criteria and compliance with the Health Insurance Portability and Accountability Act.

**Results:**

The protocol is part of a larger research project on communication and multidisciplinary collaboration in oncology units and departments spread in the Sicily region. The results of this study will particularly focus on the organization of VMTBs, involving oncology units present in different hospitals spread in the area, and creating a network to allow best patient care pathways and a hub-and-spoke relationship. The present results will also include data concerning organization skills and pitfalls, barriers, efficiency, number, and types with respect to clinical cases and customer satisfaction.

**Conclusions:**

VMTB represents a unique opportunity to optimize patient management through a patient-centered approach. An efficient virtualization and data-banking system is potentially time-saving, a source for outcome data, and a detector of possible holes in the hull of clinical pathways. The observations and results from this VMTB study may hopefully be useful to design nonclinical and organizational interventions that enhance multidisciplinary decision-making in oncology.

**International Registered Report Identifier (IRRID):**

DERR1-10.2196/26220

## Introduction

### Background

Cancer treatment represents a complex pathway that requires the collaboration of various health professionals with complementary skills who work together to share the latest evidence, pool their skills, and exchange information through a regular communication flow [1,2]. Advances in technology and the ability to customize patient treatment plans (target, molecular medical therapy, and radiotherapy) have further increased the need for regular interactions among health care professionals from different areas of expertise [3]. Consequently, in recent decades, scientific evidence has shown that cancer care has increasingly been delivered through multidisciplinary interventions by dedicated teams, the so-called multidisciplinary tumor board (MTB) [4-6].

An MTB is a team of health professionals from different clinical specialties who work together to decide the recommended best clinical pathway for an individual patient [7]. MTB members come together to discuss a series of patients to obtain a definitive staging and formulate a shared treatment plan, considering the best evidence available for personalized treatment options and appropriate follow-up [8,9]. In most cases, the multidisciplinary approach represents a useful platform for coordinating cancer care, as well as a tool for optimizing decision-making and communication processes [10,11]. As a result, MTBs improve health care delivery and the expertise for participating health professionals. Additionally, MTB participants share treatment decisions and clinical responsibility [9,12].

Despite medical literature reporting that the concept of a multidisciplinary approach to cancer treatment since 1975, MTBs in real clinical practice started in the late 1990s. From that point on, the multidisciplinary approach has continually increased, becoming a milestone in many cancer centers and a key moment in treatment plans and guidelines [13]. Over time, MTBs have evolved into a more collaborative structure with teams that pay attention to all aspects of cancer care, including rehabilitation, nutrition, psychosocial needs, and long-term care [12-16]. A few years ago, only a relatively small percentage of patients with cancer benefited from MTB-based care. Such teams currently exist for some cancers in some hospitals, but this is not the rule. Moreover, the increasing complexity of clinical pathways require a stronger interaction between high-volume centers and low-volume and community centers [13]. Before the creation of MTBs, patient evaluations were often carried out, and the oncological treatments often provided, by specialists without all the necessary knowledge and skills related to a specific tumor in terms of continuous training and adherence to local, national, and international guidelines [8,17]. The medical staff often worked in isolation owing to brief and infrequent opportunities for discussion among doctors, surgeons, radiologists, pathologists, and oncologists on the clinical, radiological, and pathological characteristics of individual cases [3,9]. Consequently, some factors relevant to decision-making were overlooked and, in some cases, patients were not considered for other treatments when these might have been useful [18].

More recently, technological advances have made collaboration between MTB members easier by introducing the possibility of “virtual” meetings when team members are not available in person [19,20]. Even if in recent years, the medical/scientific community has rightly focused on the realization of MTB, with the widespread perception that teamwork has brought benefits to patients and improved decision-making, it is necessary to focus on how MTBs function and how they will have to evolve in light of the epochal changes that SARS-CoV-2 induces in the short, medium, and long terms. Awareness of the actual provision of oncological services can help guarantee the quality of services in the face of growing demand and tight budgets through planning of actions that improve the effectiveness or efficacy of health care provision [21]. Thus, there is a need to explore new systems that allow health services professionals to access a multidisciplinary cancer treatment board regardless of their geographic location [7]. Health information technology (HIT) systems and solutions could easily solve many of the problems related to access, collection, organization, and presentation of information for MTBs, thus reducing the need for digitization of workflows [18,22].

The recent COVID-19 pandemic has augmented the necessity of reorganizing MTBs using virtual, commercially available, web-based conversation platforms [23]. Therefore, implementation of virtual multidisciplinary tumor boards (VMTBs) is a research priority which requires, regardless of technical aspects, a cultural, behavioral, and organizational change [5].

### Objectives

The aim of the project is to implement a regional wide clinical and precision medicine network and to scale the available platform to optimize its use in most common malignancies such as urogynecologic, gastrointestinal, and thoracic cancers, including breast neoplasms. Implementation of a cloud-based platform may be thwarted by physician-related barriers such as lack of time. The VMTB would support clinical decision-making, reduce unwarranted practice variation across a cancer care system, give comprehensive information about the distance between patients and a potential treatment center, and may avoid costs at hospitals lacking molecular diagnostic facilities. The VMTB would also facilitate the reporting of key statistics about each case, which will allow administrators to monitor key metrics such as improvements in time from diagnosis to treatment and the impact on patient outcomes. 

## Methods

### Study Design

The study is a pragmatic, observational, multicenter, prospective trial. The study’s programmed duration is 5 years, with a half-yearly analysis of the achievement of primary and secondary objectives.

### Study Objectives

The aim of this study is to design and implement a VMTB based on the concept of precision and molecular medicine, in the form of a retrospective and prospective observational study within the existing regional oncological care pathways. The study is aimed at allowing: (1) participation of the health professionals involved in oncology management regardless of their location, device used, and timing, so that they can provide information on cases at the best time for them; (2) participation in real-time videoconferencing from anywhere; (3) access via a wide variety of devices (phone, tablet, etc) regardless of the videoconferencing platform; and (4) identification of the most correct and efficient procedures and paths for effective development of a VMTB that can represent an interhospital network and community model.

### Primary Endpoints

As stated above, the main objectives are the feasibility and implementation of the VMTB program and acceptance of the VMTB model. Accordingly, feasibility measures include the following: (1) technical failures, defined as the inability to connect institutions; (2) technical problems, defined as equipment malfunction; (3) percentage of planned VMTB cases completed; and (4) duration of VMTB case presentations. A crucial aspect is the organization of all steps necessary to a VMTB, such as identification of participating health professionals, creation of working groups, intergroup communication, interpersonal relations, empowerment of boards, and implementation of the validated clinical pathways. The degree of adhesion of the participants to the VMTB will be measured using survey methods validated in accordance with the Delphi methodology. Each match’s degree of confidence will be measured using a 5-point Likert scale where higher scores represent more positive responses.

### Secondary Endpoints

Secondary outcomes include data on the use of the VMTB program and its effectiveness in providing access to quality and equitable cancer care, including timely and appropriate review of the multidisciplinary assessment of each case. Timely evaluation should occur within 2 weeks of the initial consultation request. Adequate multidisciplinary evaluation requires correspondence between all current oncology specialties/services and those recommended for each type of cancer in accordance with national and international guidelines (Italian Medical Oncology Association, European Society for Medical Oncology, and the National Comprehensive Cancer Network). Discussions and recommendations on each patient’s diagnosis and treatment will need to be in accordance with validated methods (Delphi or Grade) and their adherence to evidence-based medicine, national and international guidelines, or the availability of practice-changing data obtained from recently published controlled trials.

### Population and Enrollment

Participation in the project will be extended, in a nonprejudicial manner, to all the centers and professionals involved in a process with subsequent steps. The VMTB will be divided in accordance with disease types, including gynecologic cancers, urologic disease, thoracic neoplasms, and gastrointestinal tumors. Patient inclusion criteria are as follows: any patients with solid malignancy, age > 18 years, written informed consent, and processing of personal health information. Exclusion criteria are life expectancy of less than 6 months, Eastern Cooperative Oncology Group performance status > 3, and absence of informed consent and privacy. A crucial recommendation for the case presenters is to admit initially complex clinical cases, and once the VMTB is functional, to expand the presentation to all possible cases. 

### Definition of Models of Care 

#### Bulk Consulting Service

The standard method for treating cancer involves a series of specialists, one at a time. This method is least efficient as it often takes weeks or even months outside of large comprehensive cancer centers to complete visits with all consultants involved. This approach usually does not translate into the correct choice of the treatment plan. This approach may result in a nonguideline strategy or, equally negatively, the appropriate treatment sequence may be wrong. Each specialist uses his/her usual methodology throughout the patient care process. Patient satisfaction is low, as the patient travels to multiple locations multiple times and over a long time.

#### Centralized Model of Multidisciplinary Intervention (Tumor Boards)

An MTB can be a useful structure to offer integrated multi-specialist assistance. If patients present prospectively, different specialists may reach a consensus on the treatment plan and its sequence before initiating any treatment. The timeliness of care may not be solved with this approach, as patients still have to make multiple visits for an extended time.

#### Role of Telemedicine

One of the difficulties of the current case preparation process is that the information is typically contained in heterogeneous or isolated hospital databases or source systems (electronic or paper medical records, laboratory information systems, image archives, and reporting systems). The data must be collected from each network and compiled in a presentable format in anticipation of a tumor board. Professionals generally assemble this information distinctly from each other. This path creates potential communication errors, missing or duplicated information, or not using the most current information. These, and other potential workflow inefficiencies, often lead to an increase in team workload. They can also extend the time it takes to determine which treatment plan is most appropriate for a patient. The structural and functional components associated with tumor boards may also contribute to conflicting evidence and opinions regarding the impact of tumor boards on patient care or improvement in outcomes. Telemedicine has proved particularly useful for conducting multidisciplinary meetings and a solution to the downsides of standard model workflows.

### Work Teams/Analysis Units

VMTB members and their attendance at meetings depend on several factors, including the hospital’s size and the type of cancer. In general, the health professionals eligible to participate as members of VMTB are medical oncologists, radiation oncologists, surgeons, radiologists, pathologists, molecular biologists, organ or branch specialists, nurse specialists, nuclear medicine specialists, doctors of palliative medicine, general practitioners, experts in palliative care, pharmacists, and expert psychologists. Various professionals with a background in related health disciplines, such as genetic consultants, nutritionists, and plastic surgeons, may also be solicited. Finally, there may also be experts specialized in other fields relevant to the site of the tumor. Within VMTBs, identified leaders coordinate the organization of clinical services and management. Members have the level of expertise and specialization required by the MTB in question.

### Core Groups

The core groups discuss organization and implementation strategies of each VMTB. As a minimum, the core group includes a surgeon oncologist, a radiotherapist oncologist, a medical oncologist, a radiologist, and a pathologist, and a team/case manager. The core team should include any other crucial professional figure in accordance with the type of disease. There will, therefore, be a core group for each type of cancer.

### Extended Groups and Participation

In accordance with the previous statement, the VMTB may include more participants of the same categories as indicated above, who can actively participate in the discussion and drafting of each case’s minutes. Many other interested individuals can participate through organized communication. All VMTB members must include and schedule time in their work plans to prepare for and attend scheduled meetings. Core members are present for discussion of all cases where their input is required. The VMTB maintains an attendance register. Extended members and nonmembers participate in patients’ cases that are relevant to them.

### Leadership

A leader/chairperson of the VMTB and a replacement (for whenever required) need to be identified. The MTB president is responsible for organizing and running the MTB meetings. They prepare and agree over an agenda with the VMTB coordinator; ensure that the meeting agenda is appropriate and take action if not appropriate; ensure that all relevant cases are discussed and prioritized if necessary; ensure that all team members are included in the discussions; ensure that conversations are focused and relevant; ensure good communication and an environment conducive to discussion; promote evidence-based and patient-centered recommendations; ensure that the eligibility for recruitment of relevant clinical trials is considered; ensure that the patient’s current discussion and treatment/care plan recommendations are complete before discussion on the next patient begins; provide recordings of relevant demographic and clinical data; ensure that recommendations are clearly summarized, recorded, and passed on to the patient, family doctor, and clinical team within a locally agreed time period; and ensure that it is clear who will take subsequent action after the meeting while also ensuring that the meeting is recorded.

### Team Governance

Organizational support for VMTB meetings and membership are based on the premise that VMTB is the model adopted to provide effective and high-quality cancer care, with adequate funding/resources in terms of people, time, equipment, and facilities for VMTB meetings to operate effectively. Participants examine the annual assessments of MTBs and intervene on the problems that have emerged by taking appropriate improvement action.

The purpose of the VMTB and the expected results are clearly defined locally. The policies, guidelines, or protocols agree to evaluate how the MTB functions, who are the main members and extended members, the roles of the members, how members should work together, how changes in clinical practice are to be managed, and how postmeeting communications take place (ie, among patients, general practitioners, and other clinical colleagues). VMTB policies, guidelines, and protocols are reviewed at least annually. Systems are put in place for recording MTB recommendations with respect to actual treatment and warn the VMTB if treatment recommendations are not adopted, along with the underlying reasons. The VMTB regularly has the opportunity to review and take action on the experience gained in these cases and ensure that the MTB is alerted in case of serious adverse events of treatment and unexpected events/death. The MTB regularly has the opportunity to review and act on the experience gained in these cases.

### Clinical Decision-making

A set of minimum agreed upon information is provided during the meeting; that is, information that the VMTB needs to make informed recommendations, including diagnostic data (pathology and radiology), clinical information (comorbidities, psychosocial needs, and specialist and palliative care), and the patient’s medical history, points of view, and preferences. It is important that all data collected locally is digitized upon collection. VMTB considers all treatment options clinically appropriate for a patient, even those that cannot be offered or delivered locally. Case presenters have to clarify which patients should be discussed, the clinical issues to be addressed, what information must be available for the discussion to be efficacious, and when to refer a patient to another MTB.

The MTB has access to a list of all current and relevant clinical trials (including enrollment criteria) and considers patients’ eligibility for appropriate clinical trials as part of the decision-making process. Current standard treatment protocols are used whenever appropriate. The patient’s demographic profile and comorbidities are always considered. Psychosocial and supportive issues and patient palliative care are always considered. Patient views, preferences, and needs are an integral part of information during decision-making.

The clinical decision-making process translates into clear recommendations on the treatment/care plan resulting from the meeting. These recommendations ought to be evidence-based, patient-centered, in line with standard treatment protocols, or with a documented deviation. If a recommendation cannot be made owing to incomplete data or if new data become available at a later stage, it should be possible to report the patient’s case to the MTB for further discussion. MTBs collect social and clinical demographics. They review these data periodically to reflect on equal access to active treatments and other aspects of the clinical journey, care, and experience of health care professionals. 

### Virtualization and Cloud-Based Sharing

One of the difficulties of the current MTB management process is the retrieval of clinical information, which is usually found in heterogeneous hospital databases, often difficult to access, or in closed-source systems (eg, electronic medical records, laboratory information systems, image archiving and communication systems, and paper-based medical records). The data must be collected by each system and compiled in a reproducible format, in anticipation of an MTB. Doctors generally assemble this information distinctly from each other. This creates difficulties such as potential communication errors, omitted or duplicated information, or failure to use the most up-to-date information. These, and other potential inefficiencies in workflow caused by the current process of the VMTB, often lead to an increased burden on the MTB. They can also extend the time it takes to determine which treatment plan is most appropriate for a patient. The structural and functional components associated with VMTB may also contribute to conflicting evidence and opinions regarding the impact of VMTB on patient care and improvement of outcomes.

It is increasingly evident that HITs can help transform current data collection processes into more efficient and effective ones by providing the right tools.

Several HIT solutions have been analyzed in the scientific literature to improve patient data management and the workflow associated with multidisciplinary access and use. However, each of them often addresses a specific aspect of the process or deals only with 1 particular application area. Information technology solutions and strategies should easily overcome many of the difficulties of accessing, collecting, organizing, and presenting information for MTBs. In this perspective and in light of the epochal changes that the COVID-19 pandemic induces in the organizational and clinical governance processes, it is advantageous and timely to place HIT systems oriented to virtualization of meetings and cloud-based sharing of clinical information. These systems, which are already active in various research fields and medical/scientific training can now be remodeled and integrated, serving as the solution to MTB’s efficiency and efficacy bias already described. 

### Data Collection

Clinical data will be completely anonymized and will include patients’ characteristics, including demographics and disease characteristics when including oncologic and medical history, planned treatment, and clinical outcomes observed during treatments. Feasibility data related to the VMTB will record technical breakdowns and accidents, barriers to participation, pitfalls in discussion, number and type of completed planned cases, average duration of case submission, and customer satisfaction. Effectiveness relative to VMTB will consider cases that meet appropriate multidisciplinary assessment, the average time from the consultation request to the presentation of cases, and the number and percentage of cases with timely assessment within 14 days.

The data acquisition sources (ie, the ability to acquire relevant information in a timely manner) are available to the VMTB. Key information that directly affects decisions (staging, performance status, and comorbidities) is gathered by the VMTB. The data collected during the meetings are analyzed and returned to the participants to support the knowledge and learning process. The participants in internal and external audits of processes, results, and reviews audit the data (eg, to confirm that treatment recommendations correspond to current best practices and to consider recruiting test personnel), taking action to alter the practice where necessary. VMTBs consider and evaluate clinical outcome data as they become available; for example, through peer reviews and clinical target groups.

### Statistical Analysis

Statistical analyses will include descriptive statistics and comparisons made using the chi-square test or the Fisher exact test and Wilcoxon 2-sample test, as appropriate. A *P* value of <.05 will be considered significant.

### Ethics and Dissemination

This study was approved by the Ethical Commission Palermo 1, Policlinic Paolo Giaccone, University of Palermo, Italy (n°06/2020; June 24, 2020). Additional approval will be obtained from the participating organizations or oncology units accordingly to current regulations released by the Italian Agency for Medicine.

## Results

In the real world, face-to-face MTB boards are often poorly attended by many health care professionals. Several reasons may explain poor adherence to MTB such as lack of time, personal activity in several hospitals, emergencies, or familial and personal issues such as vacations. These aspects represent barriers to review all tumor cases in many hospitals or adequate participation in the MTB. Too often, these issues prevent tumor boards from reviewing all the hospital’s cancer cases or attaining full multidisciplinary participation on each case. 

New cloud-based platforms are specifically designed to facilitate VMTB functioning and therefore overcome some of the problems detected in the past, such as asynchronous participation in case discussion often limited to written chats, as previously reported [20]. On the other hand, VMTBs allow synchronous participation of as many participants as needed from different places. A particularly important aspect of VMTB concerns handling of patient data in consideration of privacy laws and regulations. Therefore, any web-based system used for VMTB must assure anonymity and secure handling of sensitive data. Many platforms currently available for web-based meetings have several built-in features if used adequately. Other possible concerns include reimbursement, interruption of workflow, and efficiency.

VMTB present several advantages since participating physicians may attend the real-time web-based meetings from any location, using a wide range of devices such as cellphones, tablets, and personal computers. VMTB-based networks may allow health professionals to participate even if they work from places distant from high-volume referral centers. These meetings also represent a unique opportunity to share cases with MTB at larger institutions or an effective teaching tool for students, residents, and newer health care professionals.

The participating hospitals were able to handle thrice as many patients through the tumor board process. Furthermore, although the number of tumor board cases tripled, they saw a higher level of participation across specialties than they did with the physical tumor board meetings. The virtual tumor board solution also gathers key statistics about each case, which will allow administrators to monitor key metrics, including improvements in time from diagnosis to treatment and the impact on patient outcomes. 

## Discussion

### Barriers

Swedish health care professionals who participated in 7 national VMTBs responded to a questionnaire that assessed key enabling factors, barriers, and opportunities for MTM development. Conventional content analysis was performed to identify thematic categories on the basis of free-text responses. Participants’ perspectives could be assigned into 3 categories: a national arena with potential for comprehensive knowledge and collaboration, prerequisites for decision-making, and organization and responsibilities. These categories consisted of 9 subcategories that referred to, for example, collective competence, resources, clinical research, case discussion, meeting climate, patient-related information, MTB potential, referrals, and technical insufficiencies [24].

A study at the Dana-Farber Cancer Institute, presented at the American Society of Clinical Oncology Annual Meeting (May 29-31, 2020), showed that physicians who are more adherent to tumor board participation are more likely to be in an academic setting, have a PhD, or navigate fewer pathways.

Tumor board preparation and session conductance need significant amount of time spent by physicians, and face-to-face MTB are usually very burdensome, often causing MTBs to fail to review all cases. Even if MTBs within high-volume centers are time-consuming and sometimes considered fastidious duty, overloaded oncologists may have difficulties in taking pace with the overwhelming increase in biomolecular knowledge. Therefore, precision medicine MTBs represent an efficient platform to stay informed and receive high-level consultations.

### Conclusions

The need for newer and fast tools to implement tumor boards is mandatory. The COVID-19 pandemic has boosted the use of virtual platforms for meetings, advisory boards, congresses, and tumor boards worldwide. However, studies reporting data on specifically designed VMTBs are very few in the medical literature.

A study carried out at Georgetown University modeled their virtual molecular tumor boards to assess the genetic makeup, previous treatment history, and other factors for 1725 patients with cancer [25]. The team compared VMTB outcomes with reviews by 5 gastrointestinal oncologists who performed tumor board duties in a conventional manner. The time spent assessing appropriate trials was noted, and the results were compared to those obtained virtually. From 2014 to 2017, researchers increased the number of patients reviewed from 46 to 622. VMTB allowed patient assessment for participation in 2000 clinical trials, use of 1000 agents, and more than 200 genetic profiles suitable for innovative treatments. Patients with pancreatic cancer represented only 5% of cases. Pishavaian et al [26] recently reported the development of a scalable, cloud-based, molecular VMTB platform, which allowed generating a treatment plan for 1725 patients, who were referred by advocacy organizations. Treatment decisions were generated in a few days on the basis of their genetic profile and a biomarker/treatment association in accordance with previous medical history, updated guidelines, and eligibility criteria for trial enrollment. This platform included a knowledge-based scoring model, rules engine, an asynchronous virtual chat room, and a reporting tool to elaborate shared and consensus reports especially for off-label treatment or clinical trial enrollment.

## Abbreviations

HIT: health information technology
MTB: multidisciplinary tumor board
VTMB: virtual multidisciplinary tumor board
